# Induced neural stem cell grafts exert neuroprotection through an interaction between Crry and Akt in a mouse model of closed head injury

**DOI:** 10.1186/s13287-021-02186-z

**Published:** 2021-02-12

**Authors:** Mou Gao, Qin Dong, Wenjia Wang, Zhijun Yang, Lili Guo, Yingzhou Lu, Boyun Ding, Lihua Chen, Jianning Zhang, Ruxiang Xu

**Affiliations:** 1 Department of Neurosurgery, Sichuan Provincial People’s Hospital, University of Electronic Science and Technology of China, Chengdu, 610072 China; 2 Department of Neurosurgery, The PLA General Hospital, Beijing, 100853 China; 3 Department of Neurology, Fu Xing Hospital, Capital Medical University, Beijing, 100038 China; 4 Department of ENT-HN, Hainan Hospital of PLA General Hospital, Sanya, 572013 China; 5 Department of Neurosurgery, The Seventh Medical Center, The PLA General Hospital, Beijing, 100700 China; 6 Department of Obstetrics, Fu Xing Hospital, Capital Medical University, Beijing, 100038 China

**Keywords:** Induced neural stem cell, Crry, Closed head injury, Transplantation, Complement

## Abstract

**Background:**

Recently, growing evidence has indicated an important role of the complement system, a crucial component of immunity, in mediating neuroinflammation and promoting neuronal apoptosis following closed head injury (CHI). We previously reported that transplanted induced neural stem cells (iNSCs) pre-treated with CHI mouse serum could enhance complement receptor type 1-related protein y (Crry) expression and ameliorate complement-mediated damage in mouse CHI models. However, the mechanism underlying the elevated levels of Crry expression remains elusive.

**Methods:**

CHI models were established using a standardized weight-drop device. We collected CHI mouse serum at 12 h post-trauma. RT-QPCR assay, western blot analysis, complement deposition assay, Akt inhibition assay, flow cytometry, cell transplantation, and functional assay were utilized to clarify the mechanism of Crry expression in iNSCs receiving CHI mouse serum treatment.

**Results:**

We observed dramatic increases in the levels of Crry expression and Akt activation in iNSCs receiving CHI mouse serum treatment. Remarkably, Akt inhibition led to the reduction of Crry expression in iNSCs. Intriguingly, the treatment of iNSC-derived neurons with recombinant complement receptor 2-conjugated Crry (CR2-Crry), which inhibits all complement pathways, substantially enhanced Crry expression and Akt activation in neurons after CHI mouse serum treatment. In subsequent in vitro experiments of pre-treatment of iNSCs with CR2-Crry, we observed significant increases in the levels of Crry expression and Akt activation in iNSCs and iNSC-derived astrocytes and neurons post-treatment with CHI mouse serum. Additionally, an in vivo study showed that intracerebral-transplanted iNSCs pre-treated with CR2-Crry markedly enhanced Crry expression in neurons and protected neurons from complement-dependent damage in the brains of CHI mice.

**Conclusion:**

INSCs receiving CR2-Crry pre-treatment increased the levels of Crry expression in iNSCs and iNSC-derived astrocytes and neurons and attenuated complement-mediated injury following CHI.

**Supplementary Information:**

The online version contains supplementary material available at 10.1186/s13287-021-02186-z.

## Introduction

Closed head injury (CHI) is regarded as a major cause of neurological disorders and impairs quality of life and creates an enormous financial burden throughout the world [1]. Following the primary mechanical trauma, secondary injury that is mainly induced by neuroinflammation has been identified as a critical factor in neuronal loss in the pathological process of CHI, leading to neurocognitive sequelae in patients [2, 3]. Recently, substantial evidence has revealed a pivotal role of the complement system in mediating neuroinflammation and promoting neuronal injury in response to CHI [4, 5]. For instance, the complement activation products C3a, C3d, C5a, and C5b-9 (membrane attack complexes), which have the potential to trigger immune cell infiltration, decrease synaptic density, and inhibit neuronal regeneration, are significantly increased in the brains of patients with traumatic head injury [6–9].

Additionally, previous studies have shown that the modulation of complement activation post-CHI by various complement regulators such as CD59, factor H, and complement receptor type 1-related protein y (Crry), which block different complement pathways, prevents ongoing neuroinflammation and neuronal damage [6–9]. For example, inhibition of C5b-9 with recombinant complement receptor 2-conjugated CD59 (CR2-CD59) ameliorates neuropathology in a mouse model of controlled cortical impact [9]. Moreover, the recombinant complement receptor 2-conjugated factor H (CR2-fH) protein, which inhibits the alternative complement pathway, can suppress neuroinflammation and improve neurological function in the treatment of CHI [8, 9].

Remarkably, brain cells can produce a certain amount of complement regulators to attenuate complement-mediated injury, but these complement regulators are markedly reduced in the brain after damage [10–12]. Furthermore, the upregulation of Crry, as shown in transgenic mice with astrocyte-targeted overexpression of Crry, can exert neuroprotective effects post-CHI [13]. Additionally, the systemic injection of Crry-Ig, a recombinant Crry molecule, enhances neuronal survival in the mouse CHI model [14]. Moreover, the administration of complement receptor 2-conjugated Crry (CR2-Crry), a recombinant fusion protein that inhibits all complement pathways, provides significant improvements in neurological functional recovery following brain damage [9].

In recent years, accumulating evidence has suggested that engrafted induced neural stem cells (iNSCs) generated from autologous somatic cells using reprogramming technology can exert beneficial effects for neuronal replacement and play positive roles in the treatment of brain injury [15, 16]. However, some investigators have argued that implanted iNSCs rarely induce a significant number of functional mature neurons [17–19]. There are many reasons for these discrepancies. For instance, inappropriate complement activation post-CHI, as mentioned above, may make iNSC-based therapy difficult because of the complement-mediated injury to iNSC grafts [20–22]. In contrast, we previously reported that systemically delivered iNSCs enhanced Crry expression and decreased neuronal apoptosis induced by complement activation in mouse CHI models [23]. Furthermore, the pre-treatment of iNSCs with CHI mouse serum as a source of active complement increased the levels of Crry expression in iNSC-derived astrocytes resulting in reduced complement-dependent damage and exerted neuroprotection following CHI [24]. However, the mechanism underlying the elevated levels of Crry expression in iNSCs and their derivatives remains poorly understood.

To clarify the mechanism of Crry expression, we performed a series of experiments and observed dramatic increases in the levels of Crry expression and Akt activation in iNSCs receiving CHI mouse serum treatment. Remarkably, Akt inhibition led to the reduction of Crry expression in iNSCs. Furthermore, after CHI mouse serum treatment, the pre-treatment of iNSCs with CHI mouse serum markedly enhanced Crry expression and Akt activation in iNSC-derived astrocytes, whereas it did not affect the levels of Crry expression or Akt activation in iNSC-derived neurons. Interestingly, we detected elevated levels of Crry expression and Akt activation in iNSC-derived neurons treated with astrocyte culture supernatants. Moreover, the treatment of iNSC-derived neurons with CR2-Crry substantially enhanced Crry expression and Akt activation in neurons following CHI mouse serum treatment. In subsequent in vitro experiments of pre-treatment of iNSCs with CR2-Crry, we observed significant increases in the levels of Crry expression and Akt activation in iNSCs and iNSC-derived astrocytes and neurons post-treatment with CHI mouse serum. Additionally, an in vivo study showed that intracerebral-transplanted iNSCs, pre-treated with CR2-Crry, markedly enhanced Crry expression in neurons and protected neurons from complement-mediated damage in the brains of CHI mice.

## Materials and methods

### CHI models

All experimental procedures were in compliance with the Guide for the Care and Use of Laboratory Animals published by the National Institutes of Health (NIH) and approved by the Committee on the Ethics of Animal Experiments of the General Hospital of Beijing Military Region, P.L.A (Permit Number: 2016-040). CHI models were established and evaluated as previously reported [25]. Detailed methods are provided in Supplementary Methods S1.

### Serum collection

CHI mouse serum was collected at 12 h post-trauma as previously described [24]. Detailed methods are provided in Supplementary Methods S2.

### Cell cultures and complement deposition assay

Cell cultures and complement deposition assay were performed as previously described [24]. Detailed methods are provided in Supplementary Methods S3.

### Akt inhibition assay

INSCs were randomly divided into three groups: the PBS, CHI, and CHI+LY294002 groups. Briefly, iNSCs were digested with Accutase (Invitrogen, Carlsbad, CA, USA) and washed with PBS (Invitrogen). The number of living cells was counted by trypan blue (Sigma-Aldrich, St. Louis, MO, USA) exclusion, and the density of the single-cell suspension was adjusted accordingly. Next, iNSCs were separately resuspended in 250 μl of PBS or CHI mouse serum in the presence or absence of LY294002 (20 μM, CST, Beverly, MA, USA) and plated onto 24-well plates (1 × 10^5^ cells per well) for 45 min at 37 °C. The cells were subsequently washed and cultured with iNSCcm for 3 days (Supplementary Methods S3). After passaging, the cells from the three groups were separately treated with CHI mouse serum for 45 min at 37 °C. Afterwards, the cells were collected and thoroughly washed.

### Cell differentiation and MACS

Cell differentiation and MACS were performed as previously described [24]. Detailed methods are provided in Supplementary Methods S4.

### Flow cytometry

Flow cytometry was performed as previously described [24]. Detailed methods are provided in Supplementary Methods S5.

### Cell viability assay

Cell viability assay was performed as previously described [24]. Detailed methods are provided in Supplementary Methods S6.

### Functional assay

Functional assay was performed as previously described [24]. Detailed methods are provided in Supplementary Methods S7.

### CR2-Crry treatment

CR2-Crry was produced and purified as previously described [26]. INSC-derived neurons were randomly divided into three groups: the PBS, CHI, and CHI+CR2-Crry groups. Briefly, neurons derived from iNSCs without any pre-treatment were separately resuspended in 250 μl of PBS or CHI mouse serum in the presence or absence of CR2-Crry (40 nM) and plated onto 24-well plates (1 × 10^5^ cells per well) for 45 min at 37 °C. Subsequently, the cells were washed and cultured with DMEM/F12 (1:1).

### Cell transplantation

Cell transplantation was performed as previously described [24]. Detailed methods are provided in Supplementary Methods S8.

### Morphological analysis

Morphological analysis was performed as previously described [24]. Detailed methods are provided in Supplementary Methods S9.

### TUNEL staining

TUNEL staining was performed as previously described [24]. Detailed methods are provided in Supplementary Methods S10.

### RT-QPCR assay

RT-QPCR assay was performed as previously described [23]. Detailed methods are provided in Supplementary Methods S11. The sequences of the PCR primer pairs used in this study were reported previously [27–29].

### Western blot analysis

Western blot analysis was performed as previously described [24]. Detailed methods are provided in Supplementary Methods S12.

### Statistical analysis

The SPSS17.0 statistical software package was used for statistical analysis. Data were presented as mean ± standard deviation (SD). Student’s *t* test and one-way ANOVA were conducted to determine statistical significance. A *P* < 0.05 was considered to be significant.

## Results

### CHI mouse serum pre-treatment led to Akt activation in iNSCs

**Fig. 1:**
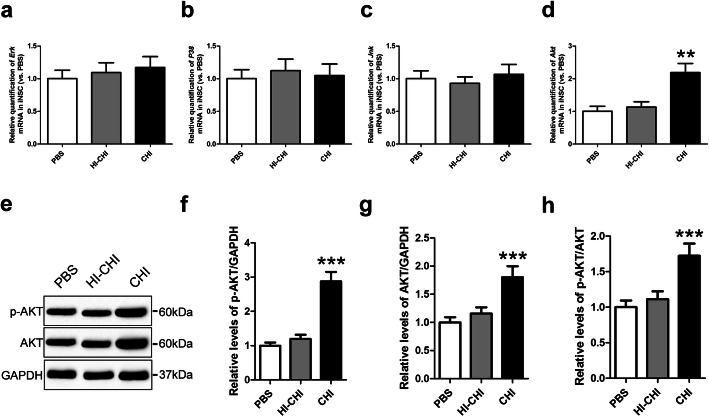
CHI mouse serum pre-treatment led to Akt activation in iNSCs. **a**–**d** RT-QPCR was utilized to determine the expression of the *Erk* (**a**), *P38* (**b**), *Jnk* (**c**), and *Akt* (**d**) genes in iNSCs among the PBS (iNSCs receiving PBS pre-treatment), HI-CHI (iNSCs receiving HI-CHI mouse serum pre-treatment), and CHI (iNSCs receiving CHI mouse serum pre-treatment) groups following treatment with CHI mouse serum for 45 min (*n* = 3/group; one-way ANOVA, **P < 0.01 versus PBS and HI-CHI groups, respectively). **e** Representative immunoblots depicting the levels of p-AKT and AKT in iNSCs among the three groups after CHI mouse serum treatment. **f**–**h** Histograms showing the relative levels of p-AKT (**f**), AKT (**g**), and p-AKT/AKT (**h**) in iNSCs among the three groups following treatment with CHI mouse serum (*n* = 6/group; one-way ANOVA, ***P < 0.001 versus PBS and HI-CHI groups, respectively)

After early CHI mouse serum treatment, we observed a dramatic increase in Crry expression in iNSCs [23, 24]. To explore the mechanism underlying this effect, we used RT-QPCR to determine the expression of the *Erk*, *P38*, *Jnk*, and *Akt* genes in iNSCs among the PBS (iNSCs receiving PBS pre-treatment), HI-CHI (iNSCs receiving HI-CHI mouse serum pre-treatment), and CHI (iNSCs receiving CHI mouse serum pre-treatment) groups following treatment with CHI mouse serum (Fig. 1a–d). The levels of the *Erk*, *P38*, and *Jnk* genes in iNSCs were not significantly different among the three groups. However, *Akt* levels in iNSCs were substantially higher in the CHI group than in the other two groups (*n* = 3/group, *P* < 0.05).

Next, we performed a western blot analysis to detect the protein expression levels of p-Akt and Akt in iNSCs among the three groups after CHI mouse serum treatment (Fig. 1e–h). The levels of p-Akt, Akt, and p-Akt/Akt in iNSCs were not significantly different between the PBS and HI-CHI groups. However, p-Akt, Akt, and p-Akt/Akt levels in iNSCs of the CHI group were markedly higher than those in the other two groups (*n* = 6/group, *P* < 0.05). These data showed that the pre-treatment of iNSCs with CHI mouse serum led to Akt activation in iNSCs.

### Akt inhibition reduced Crry expression in iNSCs pre-treated with CHI mouse serum

**Fig. 2:**
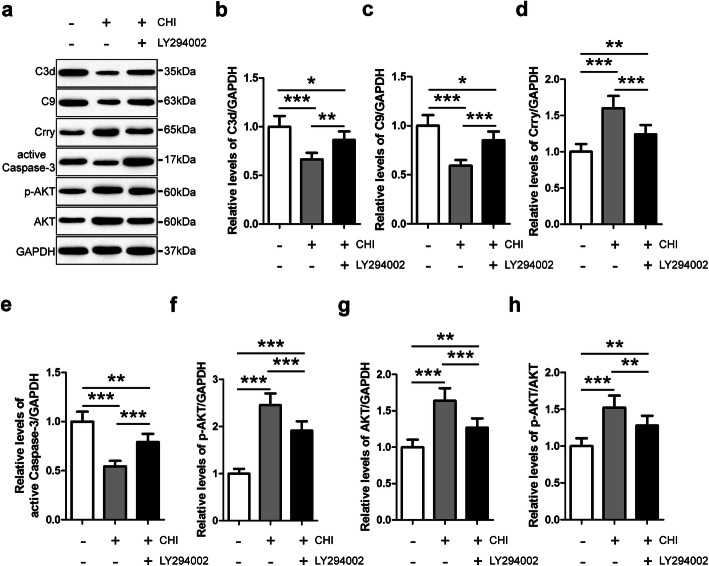
Akt inhibition reduced Crry expression in iNSCs pre-treated with CHI mouse serum. **a** Representative immunoblots depicting the levels of C3d, C9, Crry, active Caspase-3, p-AKT, and AKT in iNSCs among the PBS (iNSCs receiving PBS pre-treatment), CHI (iNSCs receiving CHI mouse serum pre-treatment), and CHI+LY294002 (iNSCs receiving CHI mouse serum and LY294002 pre-treatment) groups following treatment with CHI mouse serum for 45 min. **b**–**h** Histograms showing the relative levels of C3d (**b**), C9 (**c**), Crry (**d**), active Caspase-3 (**e**), p-AKT (**f**), AKT (**g**), and p-AKT/AKT (**h**) in iNSCs among the three groups after CHI mouse serum treatment (*n* = 6/group; one-way ANOVA, *P < 0.05, **P < 0.01, ***P < 0.001)

To examine the role of Akt signaling in mediating the immunoregulatory effect of iNSCs on complement activation, we utilized the Akt inhibitor LY294002 to pre-treat iNSCs (Fig. 2). Using western blot analysis, we observed that p-Akt, Akt, and p-Akt/Akt levels in iNSCs of the CHI+LY294002 (iNSCs receiving CHI mouse serum and LY294002 pre-treatment) group were significantly higher than those in the PBS (iNSCs receiving PBS pre-treatment) group but substantially lower than those in the CHI (iNSCs receiving CHI mouse serum pre-treatment) group following treatment with CHI mouse serum (*n* = 6/group, *P* < 0.05). Furthermore, Crry levels in iNSCs of the CHI+LY294002 group were markedly higher than those in the PBS group but significantly lower than those in the CHI group after CHI mouse serum treatment (*n* = 6/group, *P* < 0.05). In contrast, the levels of C3d, C9, and active Caspase-3 in iNSCs of the CHI+LY294002 group were substantially lower than those in the PBS group but markedly higher than those in the CHI group following treatment with CHI mouse serum (*n* = 6/group, *P* < 0.05). These findings implied that the expression of Crry in iNSCs was positively associated with the level of Akt activation in iNSCs. Moreover, Akt inhibition clearly diminished the effect of the pre-treatment of iNSCs with CHI mouse serum on Crry expression in iNSCs. Additionally, the role of Crry in the reduction of complement-mediated injury to iNSCs was significantly attenuated by Akt inhibition.

### Crry expression and Akt activation in astrocytes and neurons derived from iNSCs receiving CHI mouse serum pre-treatment

**Fig. 3:**
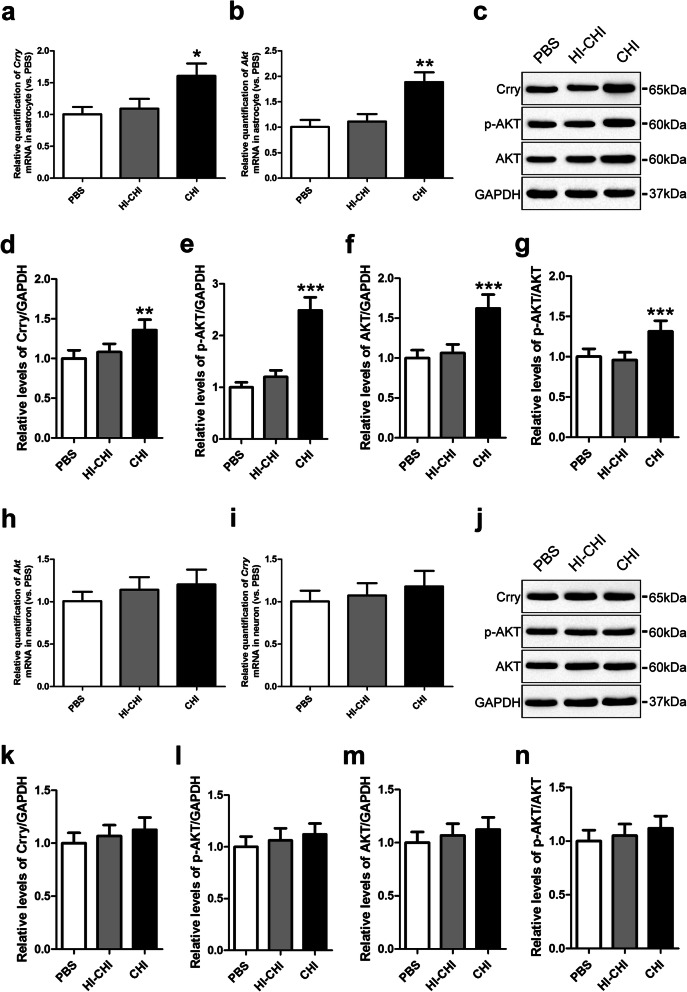
Crry expression and Akt activation in astrocytes and neurons derived from iNSCs receiving CHI mouse serum pre-treatment. **a**, **b** RT-QPCR was utilized to determine the expression of the *Crry* (**a**) and *Akt* (**b**) genes in astrocytes derived from iNSCs among the PBS (iNSCs receiving PBS pre-treatment), HI-CHI (iNSCs receiving HI-CHI mouse serum pre-treatment), and CHI (iNSCs receiving CHI mouse serum pre-treatment) groups following treatment with CHI mouse serum for 45 min (*n* = 3/group; one-way ANOVA, *P < 0.05, **P < 0.01 versus PBS and HI-CHI groups, respectively). **c** Representative immunoblots depicting the levels of Crry, p-AKT, and AKT in astrocytes derived from iNSCs among the three groups after CHI mouse serum treatment. **d**–**g** Histograms showing the relative levels of Crry (**d**), p-AKT (**e**), AKT (**f**), and p-AKT/AKT (**g**) in astrocytes derived from iNSCs among the three groups following treatment with CHI mouse serum (*n* = 6/group; one-way ANOVA, **P < 0.01, ***P < 0.001 versus PBS and HI-CHI groups, respectively). **h**, **i** The expression of the *Crry* (**h**) and *Akt* (**i**) genes in neurons derived from iNSCs among the three groups after CHI mouse serum treatment were determined by RT-QPCR (*n* = 3/group; one-way ANOVA). **j** Representative immunoblots depicting the levels of Crry, p-AKT, and AKT in neurons derived from iNSCs among the three groups following treatment with CHI mouse serum. **k**–**n** Histograms showing the relative levels of Crry (**k**), p-AKT (**l**), AKT (**m**), and p-AKT/AKT (**n**) in neurons derived from iNSCs among the three groups after CHI mouse serum treatment (*n* = 6/group; one-way ANOVA)

To evaluate the levels of Crry expression and Akt activation in iNSC-derived astrocytes, we used RT-QPCR and observed no significant differences in the levels of the *Crry* or *Akt* genes in astrocytes between the PBS (iNSCs receiving PBS pre-treatment) and HI-CHI (iNSCs receiving HI-CHI mouse serum pre-treatment) groups after CHI mouse serum treatment (Fig. 3a, b). In contrast, the levels of the *Crry* and *Akt* genes in astrocytes were substantially higher in the CHI (iNSCs receiving CHI mouse serum pre-treatment) group compared to the other two groups (*n* = 3/group, *P* < 0.05). Supporting these findings, a western blot analysis showed that the Crry, p-Akt, Akt, and p-Akt/Akt levels in astrocytes between the PBS and HI-CHI groups were almost identical, whereas the levels of Crry, p-Akt, Akt, and p-Akt/Akt in astrocytes of the CHI group were markedly higher than those in the other two groups (*n* = 6/group, *P* < 0.05) (Fig. 3c–g). These results indicated that the pre-treatment of iNSCs with CHI mouse serum enhanced Crry expression and Akt activation in iNSC-derived astrocytes.

Next, to detect Crry expression and Akt activation levels in iNSC-derived neurons, we utilized RT-QPCR and observed no significant differences in the levels of the *Crry* or *Akt* genes in neurons among the three groups after CHI mouse serum treatment (Fig. 3h, i). Furthermore, a western blot analysis also revealed that the Crry, p-Akt, Akt, and p-Akt/Akt levels in neurons among the three groups were almost identical (Fig. 3j–n). These data suggested that the pre-treatment of iNSCs with CHI mouse serum did not affect the levels of Crry expression or Akt activation in iNSC-derived neurons.

### Enhanced levels of Crry expression and Akt activation in iNSC-derived neurons treated with astrocyte culture supernatants

**Fig. 4:**
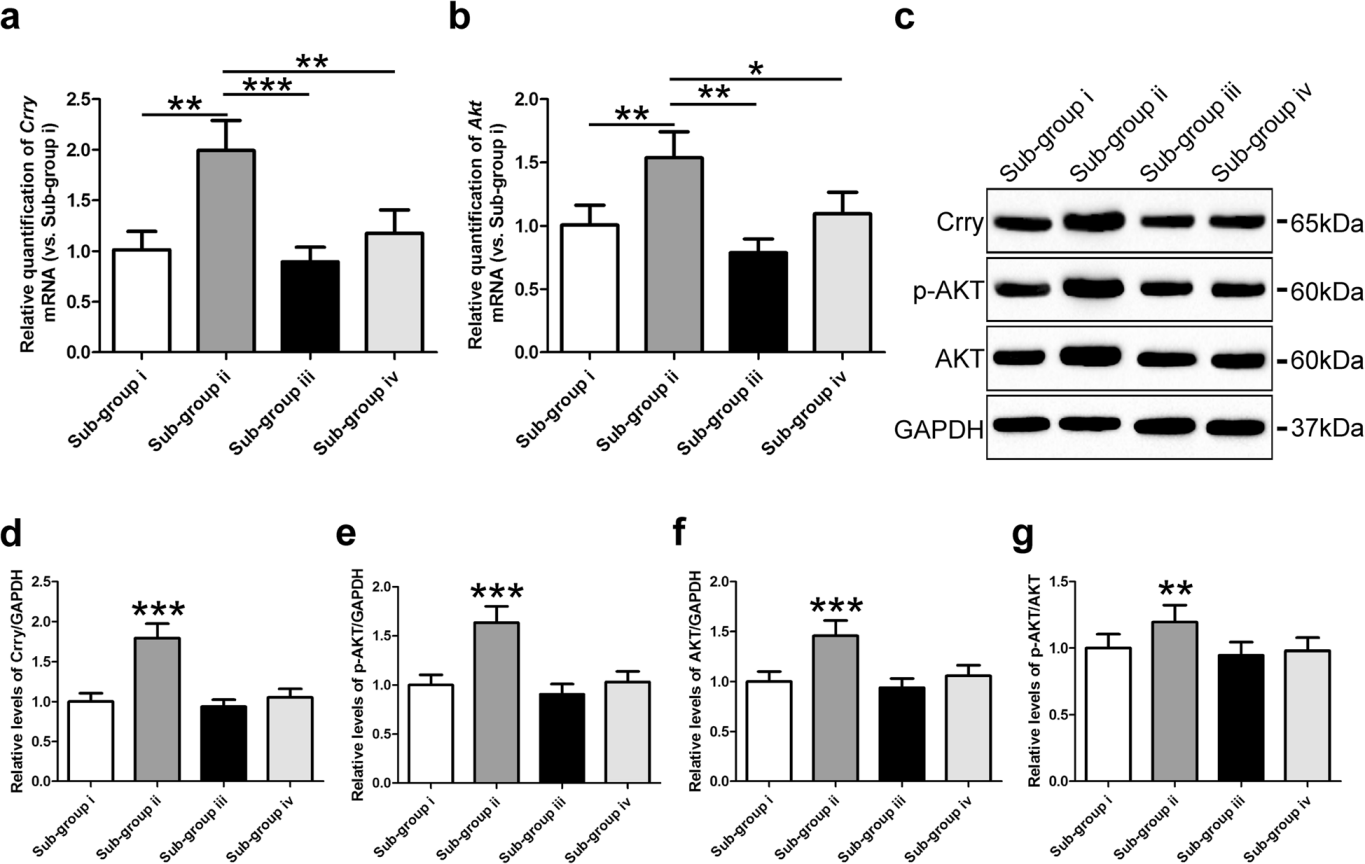
Enhanced levels of Crry expression and Akt activation in iNSC-derived neurons receiving astrocyte culture supernatants. **a**, **b** RT-QPCR was utilized to determine the expression of the *Crry* (**a**) and *Akt* (**b**) genes in neurons derived from iNSCs among the four sub-groups, which were separately treated as follows: (i) CHI mouse serum diluted (20%) in DMEM/F12 (1:1), (ii) CHI mouse serum diluted (20%) in the astrocyte culture supernatants, (iii) CHI mouse serum diluted (20%) in DMEM/F12 (1:1) containing purified rat anti-mouse Crry antibody at 5 μg ml^−1^, and (iv) CHI mouse serum diluted (20%) in the astrocyte culture supernatants containing purified rat anti-mouse Crry antibody at 5 μg ml^−1^ for 45 min at 37 °C (*n* = 3/group; one-way ANOVA, *P < 0.05, **P < 0.01, ***P < 0.001 versus i, iii, and iv sub-groups, respectively). **c** Representative immunoblots depicting the levels of Crry, p-AKT, and AKT in neurons derived from iNSCs among the four sub-groups. **d**–**g** Histograms showing the relative levels of Crry (**d**), p-AKT (**e**), AKT (**f**), and p-AKT/AKT (**g**) in neurons derived from iNSCs among the four sub-groups (*n* = 6/group; one-way ANOVA, **P < 0.01, ***P < 0.001 versus i, iii, and iv sub-groups, respectively)

We previously reported that astrocytes derived from iNSCs pre-treated with CHI mouse serum could reduce the numbers of apoptotic neurons via Crry expression following CHI mouse serum treatment [24]. To further study the mechanism underlying the neuroprotection mediated by astrocytes, we performed a functional assay and observed no significant differences in the levels of the *Crry* or *Akt* genes in neurons among the i (CHI mouse serum diluted in DMEM/F12), iii (CHI mouse serum diluted in DMEM/F12 containing purified rat anti-mouse Crry antibody), and iv (CHI mouse serum diluted in the astrocyte culture supernatants containing purified rat anti-mouse Crry antibody) sub-groups (Fig. 4a, b). However, the levels of the *Crry* and *Akt* genes in neurons were substantially higher in the ii (CHI mouse serum diluted in the astrocyte culture supernatants) sub-group than in the other three sub-groups (*n* = 3/group, *P* < 0.05). Using western blot analysis, we observed that the Crry, p-Akt, Akt, and p-Akt/Akt levels in neurons were not significantly different among the i, iii, and iv sub-groups (Fig. 4c–g). In contrast, the levels of Crry, p-Akt, Akt, and p-Akt/Akt in the neurons of the ii sub-group were markedly higher than those in the other three sub-groups (*n* = 6/group, *P* < 0.05). These findings revealed that the levels of soluble Crry in astrocyte culture supernatants were positively associated with the levels of Crry expression and Akt activation in iNSC-derived neurons.

### Crry expression and Akt activation in iNSC-derived neurons treated with CR2-Crry in the presence of CHI mouse serum

**Fig. 5:**
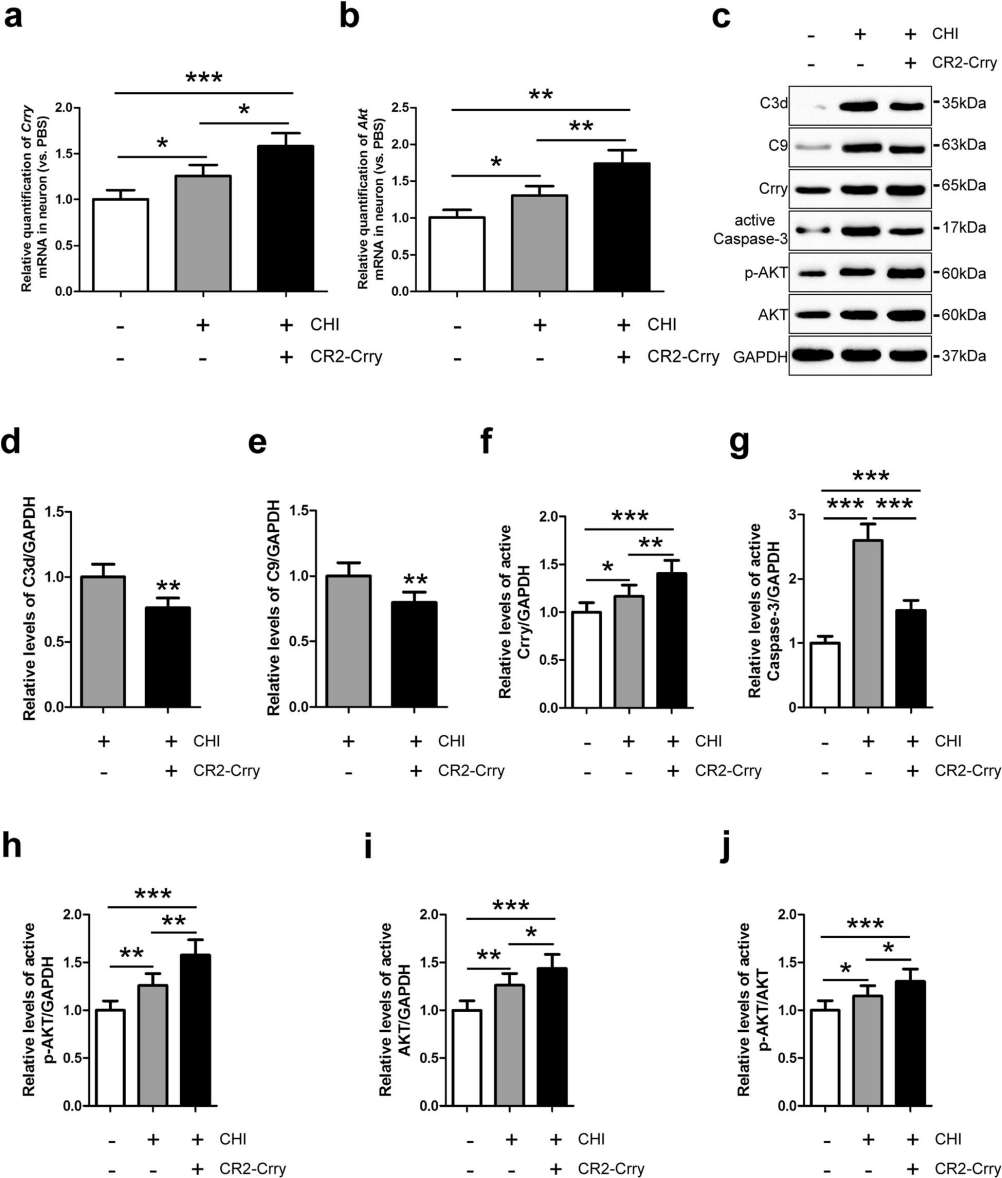
Crry expression and Akt activation in iNSC-derived neurons treated with CR2-Crry. **a**, **b** RT-QPCR was utilized to determine the expression of the *Crry* (**a**) and *Akt* (**b**) genes in neurons derived from iNSCs among the PBS (neurons receiving PBS treatment), CHI (neurons receiving CHI mouse serum treatment), and CHI+CR2-Crry (neurons receiving CHI mouse serum and CR2-Crry treatment) groups (*n* = 3/group; one-way ANOVA, *P < 0.05, **P < 0.01, ***P < 0.001). **c** Representative immunoblots depicting the levels of C3d, C9, Crry, active Caspase-3, p-AKT, and AKT in neurons derived from iNSCs among the three groups. **d**–**j** Histograms showing the relative levels of C3d (**d**), C9 (**e**), Crry (**f**), active Caspase-3 (**g**), p-AKT (**h**), AKT (**i**), and p-AKT/AKT (**j**) in neurons derived from iNSCs among the three groups (*n* = 6/group; one-way ANOVA, *P < 0.05, **P < 0.01, ***P < 0.001)

To explore the effect of Crry treatment on iNSC-derived neurons, we utilized RT-QPCR and observed that the levels of the *Crry* and *Akt* genes in neurons were substantially higher in the CHI (neurons receiving CHI mouse serum treatment) group than in the PBS (neurons receiving PBS treatment) group (*n* = 3/group, *P* < 0.05) (Fig. 5a, b). Moreover, the levels of the *Crry* and *Akt* genes in neurons were significantly higher in the CHI+CR2-Crry (neurons receiving CHI mouse serum and CR2-Crry treatment) group than in the other two groups (*n* = 3/group, *P* < 0.05). Additionally, a western blot analysis revealed that the C3d, C9, Crry, active Caspase-3, p-Akt, Akt, and p-Akt/Akt levels were low in the neurons of the PBS group (Fig. 5c–j). However, CHI mouse serum treatment induced obvious increases in the levels of C3d, C9, Crry, active Caspase-3, p-Akt, Akt, and p-Akt/Akt in the neurons of the CHI group (*n* = 6/group, *P* < 0.05). Remarkably, the Crry, p-Akt, Akt, and p-Akt/Akt levels in neurons were markedly higher in the CHI+CR2-Crry group than in the other two groups (*n* = 6/group, *P* < 0.05). Furthermore, the levels of C3d, C9, and active Caspase-3 in the neurons of the CHI+CR2-Crry group were substantially higher than those in the PBS group but significantly lower than those in the CHI group (*n* = 6/group, *P* < 0.05).

**Fig. 6:**
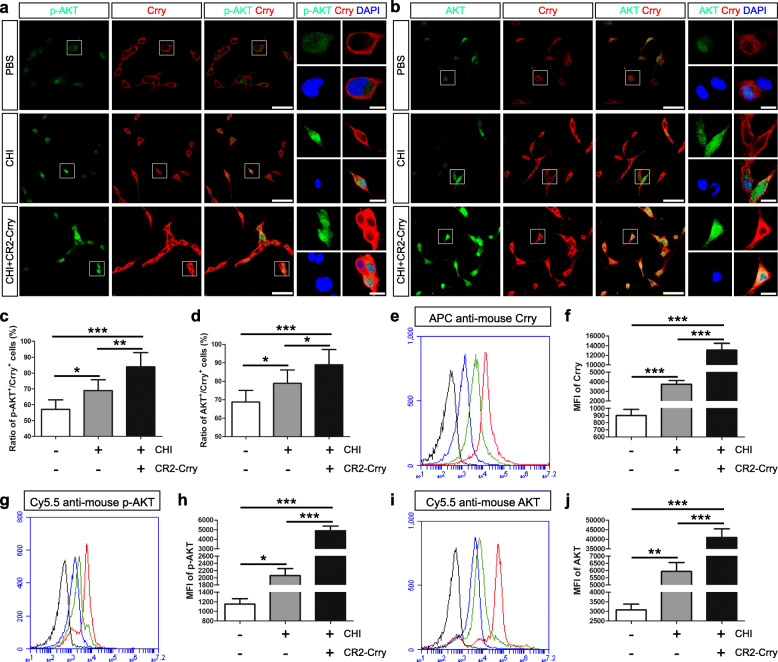
Elevated levels of Crry expression and Akt activation in iNSC-derived neurons receiving CR2-Crry treatment. **a**, **b** Representative staining for the p-AKT^+^ (green, **a**), AKT^+^ (green, **b**), and Crry^+^ (red) depicted p-AKT, AKT, and Crry levels in neurons derived from iNSCs among the PBS (neurons receiving PBS treatment), CHI (neurons receiving CHI mouse serum treatment), and CHI+CR2-Crry (neurons receiving CHI mouse serum and CR2-Crry treatment) groups. Nuclei were counterstained with DAPI (blue). **c**, **d** Histograms showing the ratio of p-AKT^+^/Crry^+^ (**c**, the number of p-AKT and Crry double-positive cells/the total number of DAPI-positive cells) and AKT^+^/Crry^+^ (**d**, the number of AKT and Crry double-positive cells/the total number of DAPI-positive cells) cells among the three groups (*n* = 6/group; one-way ANOVA, *P < 0.05, **P < 0.01, ***P < 0.001). **e**, **g**, **i** Representative flow cytometric analysis of Crry (**e**), p-AKT (**g**), and AKT (**i**) expression in neurons derived from iNSCs among the PBS (blue line), CHI (green line), and CHI+CR2-Crry (red line) groups. Isotype antibodies were used as controls (black line). **f**, **h**, **j** Histograms indicating the median fluorescence intensity (MFI) values of Crry (**f**), p-AKT (**h**), and AKT (**j**) expression in iNSC-derived neurons among the three groups (*n* = 3/group; one-way ANOVA, *P < 0.05, **P < 0.01, ***P < 0.001). Scale bar = 100 μm (15 μm)

Subsequently, we performed immunofluorescence staining and observed that the numbers of p-Akt^+^/Crry^+^ and Akt^+^/Crry^+^ neurons were markedly higher in the CHI group than in the PBS group (*n* = 6/group, *P* < 0.05) (Fig. 6a–d). Moreover, the levels of p-Akt^+^/Crry^+^ and Akt^+^/Crry^+^ neurons were substantially higher in the CHI+CR2-Crry group than in the other two groups (*n* = 6/group, *P* < 0.05). Additionally, flow cytometry analysis demonstrated that the Crry, p-Akt, and Akt levels in neurons were significantly higher in the CHI group than in the PBS group (*n* = 3/group, *P* < 0.05) (Fig. 6e–j). Furthermore, the levels of Crry, p-Akt, and Akt in neurons were markedly higher in the CHI+CR2-Crry group than in the other two groups (*n* = 3/group, *P* < 0.05). Therefore, the treatment of iNSC-derived neurons with CR2-Crry clearly enhanced Crry expression and Akt activation in neurons following CHI mouse serum treatment. Moreover, the administration of CR2-Crry effectively reduced complement-mediated injury to neurons.

### CR2-Crry pre-treatment enhanced Crry expression and Akt activation in iNSCs and iNSC-derived astrocytes and neurons

To evaluate the effect of CR2-Crry pre-treatment on iNSCs, we used RT-QPCR and observed that the levels of the *Crry* and *Akt* genes in iNSCs and iNSC-derived astrocytes and neurons were markedly higher in the CR2-Crry (iNSCs receiving CR2-Crry pre-treatment) group than in the PBS (iNSCs receiving PBS pre-treatment) group after CHI mouse serum treatment (*n* = 3/group, *P* < 0.05) (Supplementary Figures S1a, b, S2a, b, and S3a, b). In support of these findings, western blot analysis indicated that the Crry, p-Akt, Akt, and p-Akt/Akt levels in iNSCs and iNSC-derived astrocytes and neurons were substantially higher in the CR2-Crry group than in the PBS group (*n* = 6/group, *P* < 0.05) (Supplementary Figures S1c-g, S2c-g and S3c-g). Furthermore, we performed the 3-[4, 5-dimethylthiazol-2-yl]-2, 5-diphenyl tetrazolium bromide (MTT) assay to measure the viability of iNSCs and iNSC-derived astrocytes and neurons from the PBS and CR2-Crry groups following CHI mouse serum treatment (Supplementary Figures S1h, S2h and S3h). The cell viabilities between the two groups without CHI mouse serum treatment were almost identical. However, dramatic decreases in cellular viability within each group were observed after CHI mouse serum treatment (*n* = 3/group, *P* < 0.05). Moreover, the viabilities of iNSCs and iNSC-derived astrocytes and neurons were significantly lower in the PBS groups than in the CR2-Crry group (*n* = 3/group, *P* < 0.05). These data implied that, following treatment with CHI mouse serum, the pre-treatment of iNSCs with CR2-Crry clearly enhanced Crry expression and Akt activation in iNSCs and iNSC-derived astrocytes and neurons. Additionally, the pre-treatment of iNSCs with CR2-Crry markedly improved the survival of iNSCs and iNSC-derived astrocytes and neurons post-treatment with CHI mouse serum.

### Increased levels of Crry expression and Akt activation in neurons in the brains of CHI mice receiving iNSCs pre-treated with CR2-Crry

**Fig. 7:**
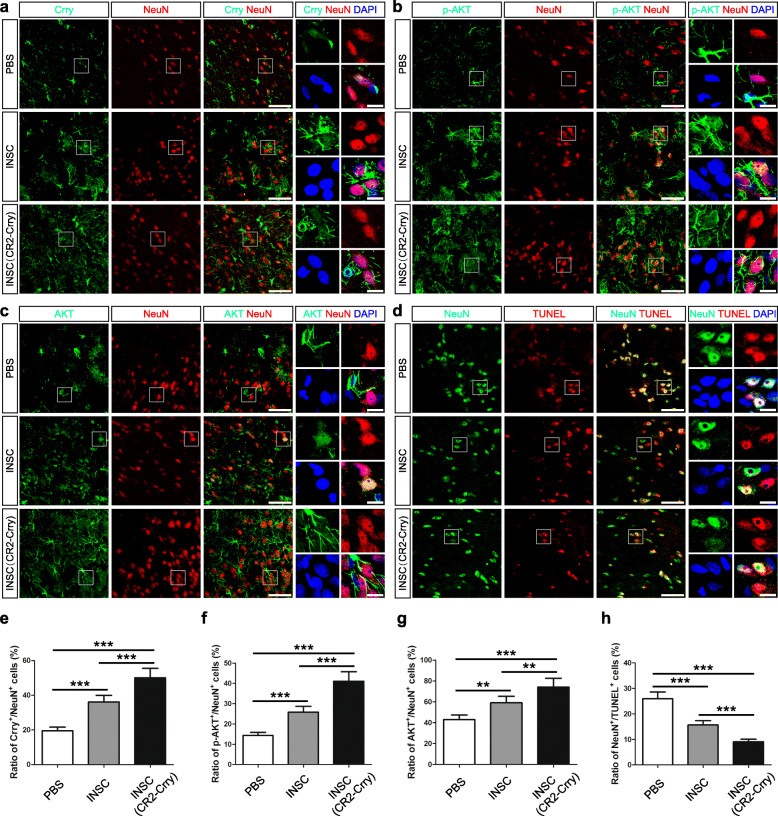
Increased levels of Crry expression and Akt activation in neurons in the brains of CHI mice receiving iNSCs pre-treated with CR2-Crry. **a**–**c** Representative staining for Crry^+^ (green, **a**), p-AKT^+^ (green, **b**), AKT^+^ (green, **c**), and NeuN^+^ (red) cells depicting the distribution of Crry^+^/NeuN^+^, p-AKT^+^/NeuN^+^, and AKT^+^/NeuN^+^ neurons in the injured cortex among the PBS (CHI mice receiving PBS), iNSC (CHI mice receiving iNSCs pre-treated with PBS), and iNSC (CR2-Crry) (CHI mice receiving iNSCs pre-treated with CR2-Crry) groups on day 14 post-CHI. Nuclei were counterstained with DAPI (blue). **d** Representative staining for NeuN^+^ (green) and TUNEL^+^ (red) cells depicting the distribution of NeuN^+^/TUNEL^+^ neurons in the injured cortex among the three groups at 14 days after CHI. Nuclei were counterstained with DAPI (blue). **e**–**h** Histograms indicating the ratio of Crry^+^/NeuN^+^ (**e**, the number of Crry and NeuN double-positive cells/the total number of DAPI-positive cells), p-AKT^+^/NeuN^+^ (**f**, the number of p-AKT and NeuN double-positive cells/the total number of DAPI-positive cells), and AKT^+^/NeuN^+^ (**g**, the number of AKT and NeuN double-positive cells/the total number of DAPI-positive cells), and NeuN^+^/TUNEL^+^ (**h**, the number of NeuN and TUNEL double-positive cells/the total number of DAPI-positive cells) cells in the injured cortex among the three groups on day 14 post-CHI (*n* = 6/group; one-way ANOVA, **P < 0.01, ***P < 0.001). Scale bar = 50 μm (5 μm)

To determine the effect of intracerebral-transplanted iNSCs receiving CR2-Crry pre-treatment on CHI mice, we performed double-labeling experiments and observed that Crry^+^/NeuN^+^, p-Akt^+^/NeuN^+^, and Akt^+^/NeuN^+^ neurons were present in the injured cortices of the iNSC (CR2-Crry; CHI mice receiving iNSCs pre-treated with CR2-Crry) group on day 14 post-trauma (Fig. 7a–c). In contrast, NeuN^+^/TUNEL^+^ neurons were evident in the injured cortices of the PBS (CHI mice receiving PBS) group at the same time point (Fig. 7d). Quantitatively, the levels of Crry^+^/NeuN^+^, p-Akt^+^/NeuN^+^, and Akt^+^/NeuN^+^ neurons were significantly higher in the iNSC (CHI mice receiving iNSCs pre-treated with PBS) group than in the PBS group (*n* = 6/group, *P* < 0.05) (Fig. 7e–g). Moreover, the numbers of Crry^+^/NeuN^+^, p-Akt^+^/NeuN^+^, and Akt^+^/NeuN^+^ neurons were markedly higher in the iNSC (CR2-Crry) group than in the other two groups (*n* = 6/group, *P* < 0.05). In addition, the levels of NeuN^+^/TUNEL^+^ neurons were substantially lower in the iNSC group than in the PBS group (*n* = 6/group, *P* < 0.05) (Fig. 7h). Furthermore, the numbers of NeuN^+^/TUNEL^+^ neurons were significantly lower in the iNSC (CR2-Crry) group than in the other two groups (*n* = 6/group, *P* < 0.05).

**Fig. 8:**
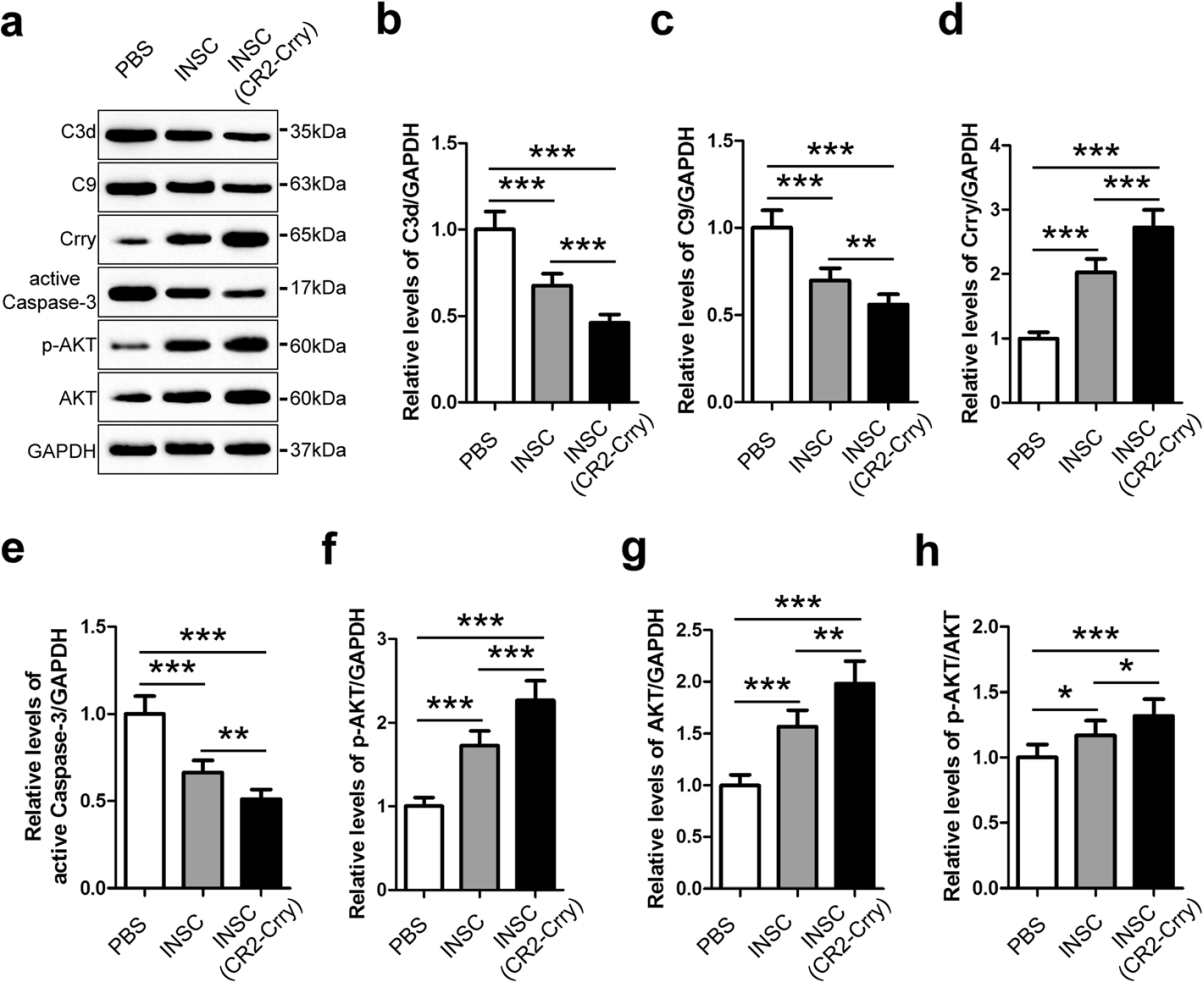
Intracerebral transplantation of iNSCs receiving CR2-Crry pre-treatment influenced Crry expression, Akt, and complement activation in the brains of CHI mice. **a** Representative immunoblots depicting the levels of C3d, C9, Crry, active Caspase-3, p-AKT, and AKT in the brains of mice among the PBS (CHI mice receiving PBS), iNSC (CHI mice receiving iNSCs pre-treated with PBS), and iNSC (CR2-Crry) (CHI mice receiving iNSCs pre-treated with CR2-Crry) groups on day 14 post-CHI. **b**–**h** Histograms showing the relative levels of C3d (**b**), C9 (**c**), Crry (**d**), active Caspase-3 (**e**), p-AKT (**f**), AKT (**g**), and p-AKT/AKT (**h**) in the brains of mice among the three groups at 14 days after CHI (*n* = 6/group; one-way ANOVA, *P < 0.05, **P < 0.01, ***P < 0.001)

Subsequently, we utilized western blot analysis to evaluate the levels of C3d, C9, Crry, active Caspase-3, p-Akt, Akt, and p-Akt/Akt in the brains of CHI mice among the three groups at 14 days after injury (Fig. 8). The levels of C3d, C9, and active Caspase-3 in the brains of the iNSC group were markedly lower than those in the PBS group (*n* = 6/group, *P* < 0.05). However, the levels of Crry, p-Akt, Akt, and p-Akt/Akt in the brains of CHI mice were substantially higher in the iNSC group than in the PBS group (*n* = 6/group, *P* < 0.05). Remarkably, the levels of C3d, C9, and active Caspase-3 in the brains of CHI mice were significantly lower, whereas the levels of Crry, p-Akt, Akt, and p-Akt/Akt were markedly higher in the iNSC (CR2-Crry) group than in the other two groups (*n* = 6/group, *P* < 0.05). In summary, intracerebral-transplanted iNSCs, pre-treated with CR2-Crry, could enhance Crry expression and Akt activation in neurons and reduce complement-mediated injury to neurons in the brains of CHI mice.

## Discussion

Neuronal apoptosis induced by complement activation following CHI is a major cause of neurological disorders [4, 5]. Growing evidence indicates that neuronal replacement using stem cell transplantation can play a positive role in the treatment of neuronal damage [15, 16]. However, complement-mediated cytolysis also exerts deleterious effects on these stem cell grafts [20–22]. We previously reported that early CHI mouse serum treatment increases the levels of Crry expression in transplanted iNSCs to reduce complement-mediated injury to grafts and hosts [23, 24]. However, the mechanism underlying the elevated levels of Crry expression remained elusive. Recent studies have suggested that Crry expression is associated with several signaling molecules, such as Erk, P38, Jnk, and Akt [30–34]. In the present study, we observed that the pre-treatment of iNSCs with CHI mouse serum significantly enhanced Crry expression and Akt activation in iNSCs. Furthermore, Akt inhibition led to the reduction of Crry expression in iNSCs. Moreover, the protective effect of Crry expression in iNSCs was substantially attenuated by Akt inhibition. Therefore, the pre-treatment of iNSCs with CHI mouse serum increased the levels of Crry expression in iNSCs mainly via Akt activation. The discrepancies between these results and previous studies, which show the roles of the Erk, P38, and Jnk signaling molecules in Crry expression, reflect the complicated effects of CHI mouse serum pre-treatment on iNSCs [30–34].

Additionally, the pre-treatment of iNSCs with CHI mouse serum markedly enhanced Crry expression and Akt activation in iNSC-derived astrocytes, whereas it did not affect the levels of Crry expression or Akt activation in iNSC-derived neurons. Furthermore, the level of Akt activation in astrocytes was significantly higher than that in neurons, consistent with previous studies showing Akt activation as a key driver of astroglial differentiation [35, 36]. Therefore, the differences in Akt activation between astrocytes and neurons may be responsible for the differences in Crry expression between them.

We previously reported that iNSC-derived astrocytes produce soluble Crry to protect neurons from complement-mediated damage [24]. In the present study, we observed that the treatment of iNSC-derived neurons with astrocyte culture supernatants substantially enhanced Crry expression and Akt activation in neurons. Furthermore, the administration of the neutralizing anti-Crry antibody showed that the levels of Crry expression and Akt activation in neurons were positively associated with the levels of Crry in astrocyte culture supernatants. These results reveal a positive effect of Crry on Akt activation, consistent with previous studies indicating Akt activation induced by Crry [32–34]. Hence, Crry in astrocyte culture supernatants may play a critical role in the increase of Crry expression in neurons through activation of Akt since Akt activation may be responsible for Crry expression.

Next, we evaluated the effect of Crry treatment on iNSC-derived neurons following complement-mediated damage. The treatment of neurons with CHI mouse serum slightly increased the levels of endogenous *Crry* and *Akt* genes in neurons. However, the levels of these genes in neurons receiving CHI mouse serum treatment were markedly lower than those in neurons receiving CHI mouse serum and CR2-Crry treatment. Additionally, western blot analysis, immunofluorescence staining, and flow cytometry demonstrated that the treatment of neurons with CHI mouse serum and CR2-Crry significantly enhanced Crry expression and Akt activation in neurons. Moreover, following CHI mouse serum treatment, the administration of CR2-Crry effectively attenuated the deposition of C3d and C9 in neurons, as well as the extent of neuronal cell death. In summary, except for the reduction of complement-mediated cytolysis mediated by CR2-Crry, the beneficial effects of CR2-Crry treatment on neuronal survival may be attributed to the elevated levels of Crry expression in neurons through Akt activation induced by CR2-Crry since Akt activation has the potential to increase the expression of Crry in neurons [11, 30, 37].

To further examine the role of CR2-Crry in neuroprotection, we performed a series of experiments and observed that the pre-treatment of iNSCs with CR2-Crry substantially enhanced Crry expression and Akt activation in iNSCs and iNSC-derived astrocytes and neurons. Furthermore, the pre-treatment of iNSCs with CR2-Crry effectively reduced the death of iNSCs and iNSC-derived astrocytes and neurons post-treatment with CHI mouse serum. These findings are partially consistent with previous research, and the differences in the levels of Crry expression and Akt activation in neurons derived from iNSCs receiving CR2-Crry pre-treatment and CHI mouse serum pre-treatment may be due to the differences in the levels of Crry between them (data not shown) [24, 30, 38]. Moreover, in addition to Crry expression in iNSC-derived astrocytes and neurons, we also observed an increase in the levels of Crry expression in oligodendrocytes derived from iNSCs receiving CR2-Crry pre-treatment, though this difference was not significant (data not shown). Therefore, subsequent work will continue to elucidate the reason behind the differences in Crry expression among these cells.

We previously reported that intracerebral-transplanted iNSCs pre-treated with CHI mouse serum clearly upregulate the levels of Crry expression in astrocytes resulting in reduced accumulation of C3d and C9 and the death of neurons in the brains of CHI mice [24]. However, it remained unclear whether iNSC grafts can enhance Crry expression in neurons in vivo. In the present study, we observed that iNSCs pre-treated with CR2-Crry significantly increased the levels of Crry expression and Akt activation in neurons in the brains of CHI mice. Moreover, grafted iNSCs receiving CR2-Crry pre-treatment substantially attenuated the deposition of C3d and C9 and the extent of neuronal cell death in injured brains. These results suggest that the pre-treatment of iNSCs with CR2-Crry can markedly enhance Crry expression in neurons and protect neurons from complement-mediated damage in the brains of CHI mice.

Taken together, these findings indicate that Crry expression in iNSCs is mainly modulated via Akt activation. Additionally, Crry has the potential to regulate the activation of Akt, which is consistent with previous studies [32–34]. Therefore, the interaction between Crry and Akt may constitute a system that leads to the constantly rising levels of Crry expression and Akt activation. The system reported here plays an important role in the reduction of complement-mediated injury to grafts and hosts post-CHI. In addition, the pre-treatment of iNSCs with CR2-Crry versus CHI mouse serum is a more convenient method that significantly increases the levels of Crry expression in iNSCs and iNSC-derived astrocytes and neurons and ameliorates complement-mediated damage in vitro and in vivo. Therefore, we will continue to examine the role of transplanted iNSCs receiving CR2-Crry pre-treatment in chronic post-traumatic neuropathology, as the complement system is implicated in both acute and chronic neuroinflammation and neurodegeneration after CHI [39–42].

## Conclusion

Our study demonstrated that iNSCs receiving CR2-Crry pre-treatment could increase the levels of Crry expression in iNSCs and iNSC-derived astrocytes and neurons and attenuate complement-mediated injury following CHI.

## Supplementary Information

**Additional file 1.**

## Data Availability

All data generated or analyzed during this study are included in this published article.

## Abbreviations

BSA: Bovine serum albumin
CHI: Closed head injury
CLSM: Confocal laser scanning microscopy
Crry: Complement receptor type 1-related protein y
CR2: Complement receptor 2
FBS: Fetal bovine serum
FC: Flow cytometry
IF: Immunofluorescence
INSCs: Induced neural stem cells
MTT: 3-[4, 5-Dimethylthiazol-2-yl]-2, 5-diphenyl tetrazolium bromide
NSS: Neurological severity score
PFA: Paraformaldehyde
PLL: Poly-l-lysine
RT: Room temperature
RT-QPCR: Quantitative real-time polymerase chain reaction
WB: Western blot
SPSS: Statistics Package for Social Science
