# Supplementary material for: Induced neural stem cell grafts exert neuroprotection through an interaction between Crry and Akt in a mouse model of closed head injury
Source: Stem Cell Res Ther. 2021 Feb 12;12:128. doi: 10.1186/s13287-021-02186-z (PMC7881465; doi:10.1186/s13287-021-02186-z)
Supplement: Supplementary file 1 — Additional file 1. [file 13287_2021_2186_MOESM1_ESM.doc]

**Induced neural stem cell grafts exert neuroprotection through an interaction between Crry and Akt in a mouse model of closed head injury**

Running Title: **INSCs pre-treated with CR2-Crry**

Mou Gao^1,2†^, Qin Dong^3†^, Wenjia Wang^4†^, Zhijun Yang^5†^, Lili Guo^1†^, Yingzhou Lu^6^, Boyun Ding^5^, Lihua Chen^1*^, Jianning Zhang^2*^ and Ruxiang Xu^1,5*^

^*^Correspondence: jzprofxu@126.com (Ruxiang Xu); jnzhang2018@163.com (Jianning Zhang); zljp824gk@163.com (Lihua Chen)

^†^Mou Gao, Qin Dong, Wenjia Wang, Zhijun Yang and Lili Guo contributed equally to this work.

^1^Department of Neurosurgery, Sichuan Academy of Medical Sciences and Sichuan Provincial People’s Hospital, School of Medicine, University of Electronic Science and Technology of China, Chengdu 610072, China. ^2^Department of Neurosurgery, The PLA General Hospital, Beijing 100853, China. ^3^Department of Neurology, Fu Xing Hospital, Capital Medical University, Beijing 100038, China. ^4^Department of ENT-HN, Hainan Hospital of PLA General Hospital, Sanya 572013, China. ^5^Department of Neurosurgery, The Seventh Medical Center, The PLA General Hospital, Beijing 100700, China. ^6^Department of Obstetrics, Fu Xing Hospital, Capital Medical University, Beijing 100038, China.


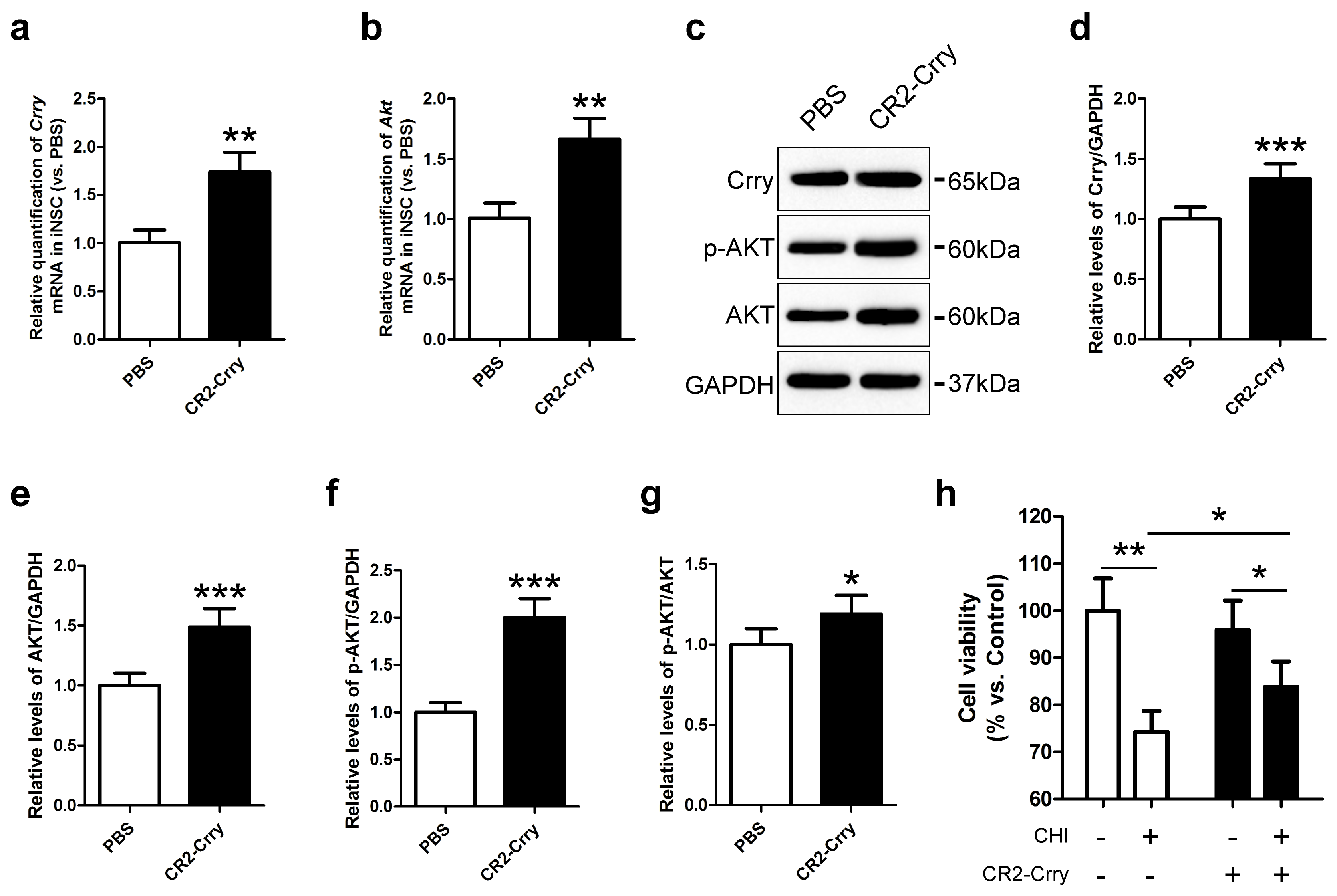


**Supplementary Fig. S1** CR2-Crry pre-treatment enhanced Crry expression and Akt activation in iNSCs. (**a**, **b**) RT-QPCR was utilized to determine the expression of the *Crry* (**a**), and *Akt* (**b**) genes in iNSCs between the PBS (iNSCs receiving PBS pre-treatment), and CR2-Crry (iNSCs receiving 40 nM CR2-Crry pre-treatment for 45 min at 37°C) groups following treatment with CHI mouse serum for 45 min (n=3/group; Student’s *t*-test (Independent-Samples T Test), ***P*<0.01 versus PBS group). (**c**) Representative immunoblots depicting the levels of Crry, p-AKT and AKT in iNSCs between the two groups after CHI mouse serum treatment. (**d**-**g**) Histograms showing the relative levels of Crry (**d**), p-AKT (**e**), AKT (**f**), and p-AKT/AKT (**g**) in iNSCs between the two groups following treatment with CHI mouse serum (n=6/group; Student’s *t*-test (Independent-Samples T Test), **P*<0.05, ****P*<0.001 versus PBS group). (**f**) Cells from the two groups were treated with CHI mouse serum for 45 min. Subsequently, cell viability was detected using an MTT assay. The viability of cells in the PBS group without CHI mouse serum treatment was considered to be at 100% (n=3/group; Student’s *t*-test (Paired-Samples T Test), **P*<0.05, ***P*<0.01 versus PBS and CR2-Crry groups without CHI mouse serum treatment, respectively; Student’s *t*-test (Independent-Samples T Test), **P*<0.05 versus PBS group post-treatment with CHI mouse serum).


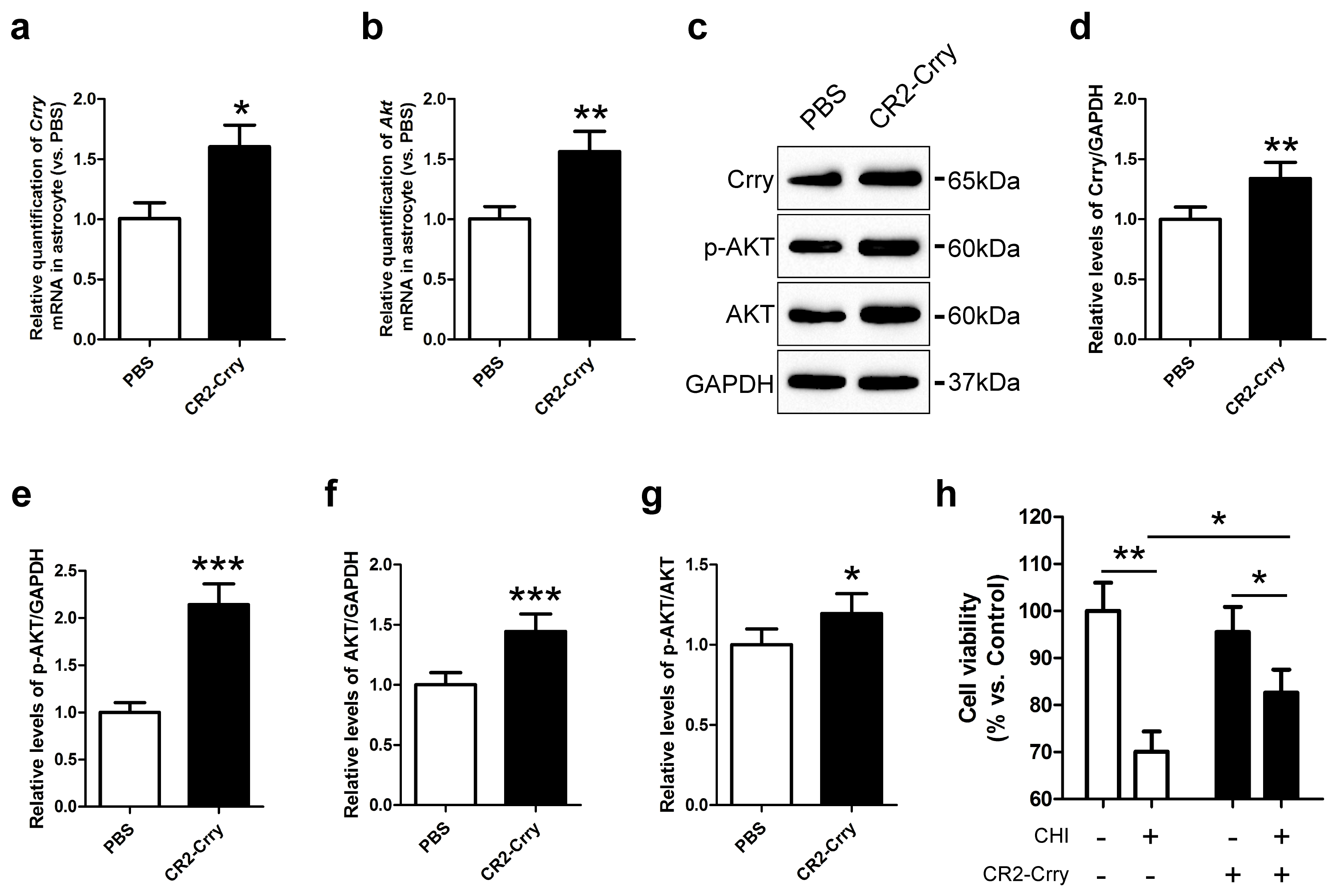


**Supplementary Fig. S2** CR2-Crry pre-treatment enhanced Crry expression and Akt activation in iNSC-derived astrocytes. (**a**, **b**) RT-QPCR was utilized to determine the expression of the *Crry* (**a**), and *Akt* (**b**) genes in astrocytes derived from iNSCs between the PBS (iNSCs receiving PBS pre-treatment), and CR2-Crry (iNSCs receiving 40 nM CR2-Crry pre-treatment for 45 min at 37°C) groups following treatment with CHI mouse serum for 45 min (n=3/group; Student’s *t*-test (Independent-Samples T Test), **P*<0.05, ***P*<0.01 versus PBS group). (**c**) Representative immunoblots depicting the levels of Crry, p-AKT and AKT in astrocytes derived from iNSCs between the two groups after CHI mouse serum treatment. (**d**-**g**) Histograms showing the relative levels of Crry (**d**), p-AKT (**e**), AKT (**f**), and p-AKT/AKT (**g**) in astrocytes derived from iNSCs between the two groups following treatment with CHI mouse serum (n=6/group; Student’s *t*-test (Independent-Samples T Test), **P*<0.05, ***P*<0.01, ****P*<0.001 versus PBS group). (**f**) Cells from the two groups were treated with CHI mouse serum for 45 min. Subsequently, cell viability was detected using an MTT assay. The viability of cells in the PBS group without CHI mouse serum treatment was considered to be at 100% (n=3/group; Student’s *t*-test (Paired-Samples T Test), **P*<0.05, ***P*<0.01 versus PBS and CR2-Crry groups without CHI mouse serum treatment, respectively; Student’s *t*-test (Independent-Samples T Test), **P*<0.05 versus PBS group post-treatment with CHI mouse serum).


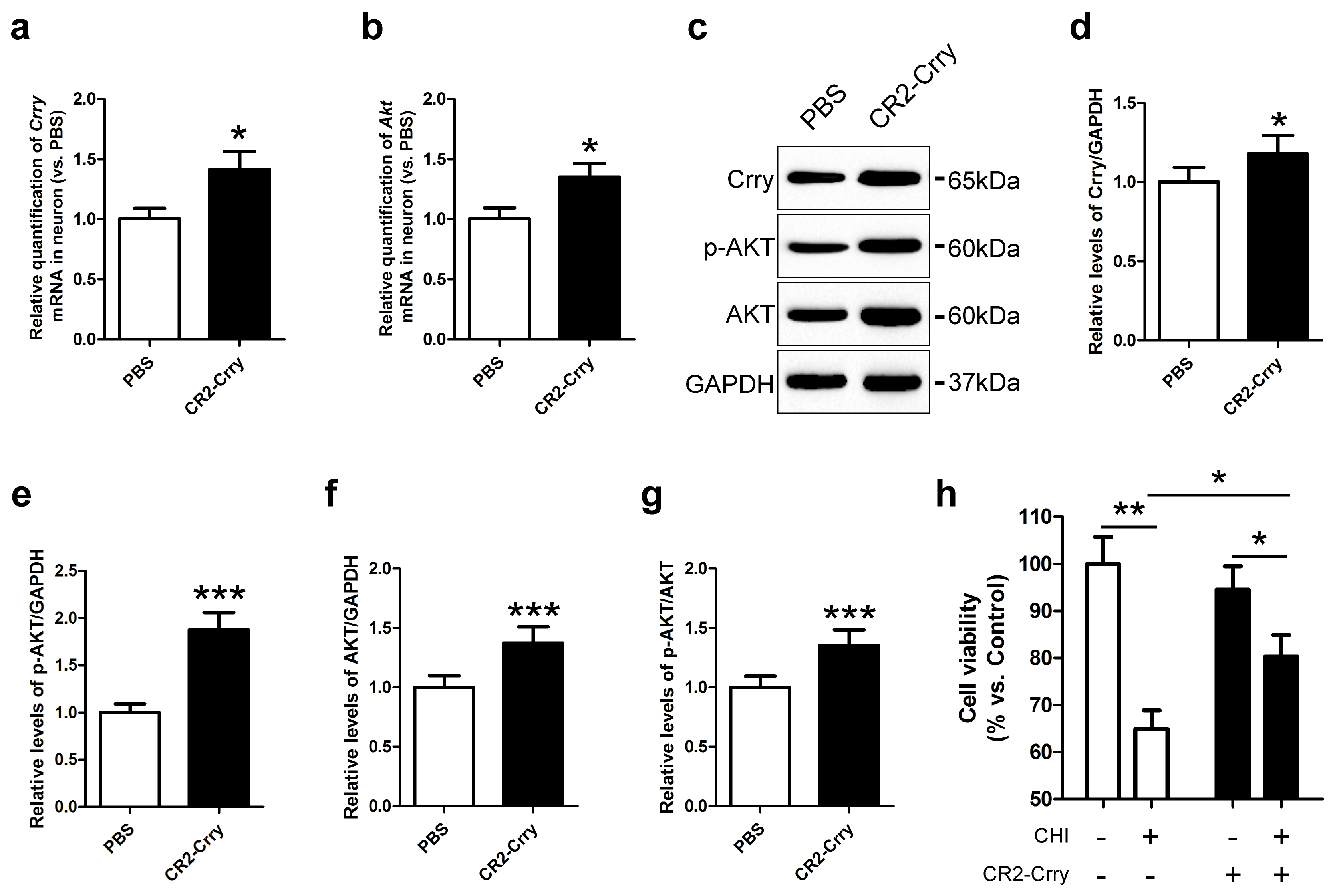


**Supplementary Fig. S3** CR2-Crry pre-treatment enhanced Crry expression and Akt activation in iNSC-derived neurons. (**a**, **b**) RT-QPCR was utilized to determine the expression of the *Crry* (**a**), and *Akt* (**b**) genes in neurons derived from iNSCs between the PBS (iNSCs receiving PBS treatment), and CR2-Crry (iNSCs receiving 40 nM CR2-Crry pre-treatment for 45 min at 37°C) groups following treatment with CHI mouse serum for 45 min (n=3/group; Student’s *t*-test (Independent-Samples T Test), **P*<0.05 versus PBS group). (**c**) Representative immunoblots depicting the levels of Crry, p-AKT and AKT in neurons derived from iNSCs between the two groups after CHI mouse serum treatment. (**d**-**g**) Histograms showing the relative levels of Crry (**d**), p-AKT (**e**), AKT (**f**), and p-AKT/AKT (**g**) in neurons derived from iNSCs between the two groups following treatment with CHI mouse serum (n=6/group; Student’s *t*-test (Independent-Samples T Test), **P*<0.05, ****P*<0.001 versus PBS group). (**f**) Cells from the two groups were treated with CHI mouse serum for 45 min. Subsequently, cell viability was detected using an MTT assay. The viability of cells in the PBS group without CHI mouse serum treatment was considered to be at 100% (n=3/group; Student’s *t*-test (Paired-Samples T Test), **P*<0.05, ***P*<0.01 versus PBS and CR2-Crry groups without CHI mouse serum treatment, respectively; Student’s *t*-test (Independent-Samples T Test), **P*<0.05 versus PBS group post-treatment with CHI mouse serum).

**Supplementary Methods S1: CHI models.** Healthy adult male C57BL/6 (B6) mice weighing 24-30 g (Vital River Laboratories, Beijing, China) were housed in a temperature- and humidity-controlled room with food and water *ad libitum*. All experimental procedures were in compliance with the Guide for the Care and Use of Laboratory Animals published by the National Institutes of Health (NIH) and approved by the Committee on the Ethics of Animal Experiments of the General Hospital of Beijing Military Region, P.L.A (Permit Number: 2016-040). Animals were anaesthetized through the intranasal administration of isoflurane (induction: 3% isoflurane; maintenance: 1.25% isoflurane) and received fentanyl (0.05 mg kg^-1^ body weight per day, intraperitoneal injection) as the analgesic agent. The parietal bone was exposed by a midline scalp incision after shaving and cleaning the skin. A free-falling rod with a blunt tip of 3.0 mm diameter was dropped onto the mouse’s skull (2.0 mm anterior to the lambda suture and 2.0 mm lateral to the middle line) at a falling height of 3.0 cm. Subsequently, the scalp wound was sutured and treated with povidone-iodine solution. After surgery, the mice were allowed to recover on a heating pad until fully awake. Sham-operated mice underwent the same procedures (anaesthesia, analgesia, and scalp incision), but not head trauma. Two blinded, trained investigators evaluated the animals at 1 h post-CHI using a neurological severity score (NSS). Mice with an NSS of 4-8 were enrolled in the present study.

**Supplementary Methods S2: Serum collection.** Blood, harvested via cardiac puncture, was transferred to sterile BD Vacutainer SST^TM^ tubes (BD Biosciences, San Jose, CA, USA) and subsequently centrifuged at 1690 g for 20 min at 4°C (Sorvall ST 16R, Thermo Scientific, Hudson, NH, USA). The supernatants were collected and stored at -80°C. HI-CHI (heat-inactivated CHI) mouse serum was processed by heating to 56°C for 45 min.

**Supplementary Methods S3: Cell cultures and complement deposition assay.** B6 mouse GFP-expressing iNSCs were plated at a density of 5x10^5^ cells/mL in iNSC culture medium (iNSCcm, Neurobasal: DMEM/F12 (1:1) containing 2% B27 supplements, 20 ng/ml bFGF, 20 ng/ml EGF, 0.05% bovine serum albumin (BSA) and 2 mM L-glutamine) (Invitrogen, Carlsbad, CA, USA).

For complement deposition assay, iNSCs were randomly divided into three groups: the PBS, HI-CHI and CHI groups. Briefly, iNSCs were digested with Accutase (Invitrogen) and washed with PBS (Invitrogen). The number of living cells was counted by trypan blue (Sigma-Aldrich, St. Louis, MO, USA) exclusion, and the density of the single-cell suspension was adjusted accordingly. Next, iNSCs were separately resuspended in 250 μl of PBS, HI-CHI or CHI mouse serum, and plated onto 24-well plates (1x10^5^ cells per well) for 45 min at 37°C. Subsequently, the cells were washed and cultured with iNSCcm for 3 days. After passaging, the cells from the three groups were separately treated with CHI mouse serum for 45 min at 37°C. Afterwards, the cells were collected and thoroughly washed.

**Supplementary Methods S4: Cell differentiation and MACS.** For differentiation assay, iNSCs from the PBS, HI-CHI and CHI groups were respectively plated onto poly-l-lysine- (PLL, Sigma-Aldrich) coated 24-well plates (5x10^4^ cells per well, Sigma-Aldrich) in DMEM/F12 (1:1) (Invitrogen) supplemented with 2% B27 (Invitrogen) and 0.5% foetal bovine serum (FBS, Invitrogen) for 7 days.

After 7 days of differentiation, the cells were dissociated into single-cell suspensions using Accutase. Neurons were enriched using the Neuron Isolation Kit (Miltenyi Biotec Inc., Auburn, CA, USA) by depletion of non-neuronal cells using the MACS technique, according to the manufacturer’s recommendations. Briefly, non-neuronal cells were indirectly magnetically labelled with biotin-conjugated antibodies and Anti-Biotin MicroBeads (Miltenyi Biotec Inc.). Subsequently, the magnetically labelled non-neuronal cells were retained within a MACS Column, which was placed in the magnetic field of a MACS Separator (Miltenyi Biotec Inc.), while the unlabelled neurons ran through. Furthermore, astrocytes and oligodendrocytes were respectively enriched using the anti-GLAST (ACSA-1) MicroBead Kit and anti-O4 MicroBeads (Miltenyi Biotec Inc.) according to the manufacturer’s instructions. After MACS enrichment, neurons, astrocytes and oligodendrocytes were counted using flow cytometry and immunofluorescence staining for analysis of purity and identity.

**Supplementary Methods S5: Flow** **cytometry.** Cultured cells were fixed in 4% paraformaldehyde (PFA) in 0.1 M PBS (PH 7.4) for 10 min and subsequently blocked with 10% bovine serum albumin (BSA)/0.3% TritonX-100 for 20 min on ice. The cells were subsequently incubated with primary antibodies (Supplementary Table 1) for 30 min at 4°C. After washing with PBS, the cells were incubated for 30 min at room temperature (RT) with secondary antibodies (Supplementary Table 1). After several washes, the cells were resuspended in PBS and analysed on an Accuri C6 Flow Cytometer System (BD Biosciences). Isotype control antibodies (Supplementary Table 1) were used at the same concentrations.

**Supplementary Methods S6: Cell viability assay.** Cell viability was measured using an MTT (Sigma-Aldrich, St. Louis, MO, USA) assay according to the manufacturer’s instructions. The net absorbance from the plated cells in the PBS group without CHI mouse serum treatment was considered to be at 100% cell viability.

**Supplementary Methods S7: Functional assay.** First, following treatment with CHI mouse serum, the astrocyte culture supernatants in the CHI group were collected and purified by centrifugation for 20 min at 1690 g. Second, neurons derived from iNSCs in the CHI group were randomly divided into four sub-groups and separately treated as follows: (i) CHI mouse serum diluted (20%) in DMEM/F12 (1:1); (ii) CHI mouse serum diluted (20%) in the astrocyte culture supernatants; (iii) CHI mouse serum diluted (20%) in DMEM/F12 (1:1) containing purified rat anti-mouse Crry antibody at 5 μg ml^-1^; and (iv) CHI mouse serum diluted (20%) in the astrocyte culture supernatants containing purified rat anti-mouse Crry antibody at 5 μg ml^-1^ for 45 min at 37°C. Subsequently, the neurons were collected for morphological and molecular biological analyses.

**Supplementary Methods S8: Cell transplantation.** Following pre-treatment with PBS or CR2-Crry at 37°C (45 min), iNSCs were harvested and thoroughly washed for transplantation assay. The number of living cells was counted, and the density of the single-cell suspension was adjusted as described above. Subsequently, the cells were maintained on ice. At 12 h after CHI, the mice were anaesthetized again and mounted in a stereotaxic apparatus (Stoelting, Wood Dale, IL, USA). Cell suspension or PBS was separately injected into the brain (motor cortex, 5.0 mm anterior to the lambda suture, 1.0 mm lateral to the middle line and 2.0 mm under the dura) via different sterile 25-μl 22 S Hamilton syringes. Each site received 5 μl of cell suspension containing 1x10^6^ cells or PBS at a speed of 0.5 μl min^-1^. Approximately 5 min after injection, the syringe was slowly withdrawn. At the indicated time points post-CHI, the animals were sacrificed after anaesthesia and their fresh or perfused-fixed brain tissues were collected for morphological and molecular biological analyses.

**Supplementary Methods S9: Morphological analysis.** Brain tissues were postfixed in 4% PFA in 0.1 M PBS (PH 7.4) at 4°C overnight and subsequently sectioned (10 μm) on a cryostat (Leica CM 1950, Leica Biosystems, Nussloch, Germany) and mounted on adhesion microscope slides. For immunofluorescence, slides of brain tissues and cultured cells were blocked for 1 h using 10% BSA/0.3% TritonX-100 and subsequently incubated overnight at 4°C with primary antibodies (Supplementary Table 1). After washing in PBS, the cells were incubated for 1-2 h at RT with secondary antibodies (Supplementary Table 1). After several washes with PBS, the nuclei were stained with DAPI Fluoromount-G (SouthernBiotech, Birmingham, AL, USA), and staining was detected via fluorescence microscopy (DM3000, Leica) and CLSM (TCS SP5 II, Leica). The number of positive cells was manually counted via microscopy at 20x magnification and adjusted using image analysis software (Image-Pro plus 5.0). The ratio of positive cells was calculated as (the number of positive cells/the total number of DAPI-positive cells) x100%.

**Supplementary Methods S10: TUNEL staining.** TUNEL staining was performed using the In Situ Cell Death Detection Kit with TMR red (Roche, Mannheim, Germany) according to the manufacturer’s instructions. The nuclei were counterstained with DAPI Fluoromount-G, and staining was detected via fluorescence microscopy. The ratio of TUNEL-positive cells was calculated as (the number of TUNEL-positive cells/the total number of DAPI-positive cells) x100%.

**Supplementary Methods S11: RT-QPCR assay.** RNA extracted from cultured cells was reversely transcribed into cDNA using the QuantScript RT kit (Tiangen Biotech, Beijing, China) and assessed by RT-QPCR assay using the SYBR-Green Master Mix (TaKaRa Biotech, Dalian, China) and a ViiA7 Real-Time PCR System (Applied Biosystems, Foster City, CA, USA).

**Supplementary Methods S12: Western blot analysis.** Protein was extracted from cultured cells and brain tissues using the RIPA reagent (Sigma-Aldrich) supplemented with protease and phosphatase inhibitors (Fermentas, Burlington, Canada). Protein concentrations were determined using the BCA assay (Thermo Scientific). Protein samples were heated for 10 min at 95°C, separated using SDS-PAGE (35 μg per lane), and transferred to PVDF membranes (Millipore, Bedford, MA, USA). The blots were blocked for 1 h at RT with 5% BSA in TBST and subsequently detected using incubation with primary antibodies (Supplementary Table 1) at 4°C overnight. After several washes, the blots were incubated for 1 h at RT with HRP-conjugated secondary antibodies (Supplementary Table 1). Immunoblots were visualized using the SuperSignal ECL (Pierce, Rockford, IL, USA). The results were expressed relative to the control and normalized to GAPDH.

| Specificity | Host | Detection | Concentration | Application | Origin |
| --- | --- | --- | --- | --- | --- |
| Akt (60 kDa) | Rabbit | INSC/Astrocyte/  Neuron/Brain | 0.34 μg/ml | WB/IF/FC | CST |
| Phospho-Akt (Ser473) (60 kDa) | Rabbit | INSC/Astrocyte/  Neuron/Brain | 0.20 μg/ml | WB/IF/FC | CST |
| C3d (35 kDa) | Goat | INSC/Neuron/Brain | 5 μg/ml | WB | R&D Systems |
| C9 (63 kDa) | Rabbit | INSC/Neuron/Brain | 1 μg/ml | WB | Abcam |
| Crry (65 kDa) | Rat | INSC/Astrocyte/  Neuron/Brain | 5 μg/ml | WB/IF/FC | BD Biosciences |
| Cleaved Caspase-3  (17 kDa) | Rabbit | INSC/Neuron/Brain | 1 μg/ml | WB | Abcam |
| GAPDH  (37 kDa) | Rabbit | INSC/Astrocyte/  Neuron/Brain | 0.2 μg/ml | WB | Santa Cruz |
| Rabbit IgG | Goat | Akt/Phospho-Akt (Ser473)/ C9/Active Caspase-3/ GAPDH | 0.08 μg/ml | WB | ZSGB-BIO |
| Rat IgG | Goat | Crry | 0.08 μg/ml | WB | ZSGB-BIO |
| Goat IgG | Rabbit | C3d | 0.08 μg/ml | WB | ZSGB-BIO |
| NeuN | Mouse | Brain | 5 μg/ml | IF | Millipore |
| Rabbit IgG | Alexa Fluor® 555 Goat | Akt/Phospho-Akt (Ser473) | 2 μg/ml | IF | Life Tech |
| Rat IgG | Alexa Fluor® 633 Goat | Crry (Neuron) | 2 μg/ml | IF | Life Tech |
| Rat IgG | Alexa Fluor® 555 Goat | Crry (Brain) | 2 μg/ml | IF | Life Tech |
| Mouse IgG | Alexa Fluor® 633 Goat | NeuN | 2 μg/ml | IF | Life Tech |
| Rat IgG2α, κ Isotype Control | | | 5 μg/ml | FC | BD Biosciences |
| APC Goat Anti-Rat IgG | | Crry/Rat IgG2α, κ Isotype Control | 2 μg/ml | FC | BD Biosciences |
| Rabbit IgG, monoclonal Isotype Control | | | 5 μg/ml | FC | Abcam |
| Cy5.5 Goat Anti-Rabbit IgG | | Akt/Phospho-Akt (Ser473)/  Rabbit IgG, monoclonal Isotype Control | 5 μg/ml | FC | Abcam |

**Supplementary Table 1.** Antibodies were used in this study (WB: Western blot; IF: Immunofluorescence; FC: Flow cytometry; CST, Beverly, MA, USA; R&D Systems, Minneapolis, MN, USA; Abcam, Cambridge, MA, USA; BD Biosciences, San Jose, CA, USA; Santa Cruz, Santa Cruz, CA, USA; ZSGB-BIO, Beijing, China; Millipore, Bedford, MA, USA; Life Tech, Gaithersburg, MD, USA).
